# The hidden role of heterotrophic bacteria in early carbonate diagenesis

**DOI:** 10.1038/s41598-024-84407-y

**Published:** 2025-01-02

**Authors:** Mónica Sánchez-Román, Viswasanthi Chandra, Sebastian Mulder, Camila Areias, John Reijmer, Volker Vahrenkamp

**Affiliations:** 1 Geobiology Laboratory, Earth Sciences Department, Faculty of Science, Vrije Universiteit, Amsterdam, The Netherlands; 2 Ali I. Al-Naimi Petroleum Engineering Research Center, King Abdullah University of Science and Technology, Thuwal, Saudi Arabia; 3 Saudi Aramco, Dhahran, Saudi Arabia; 4 Geo-Energy, Energy and Sustainability Research Institute Groningen, Faculty of Science and Engineering, University of Groningen, Groningen, The Netherlands

**Keywords:** Microbiology, Biogeochemistry, Environmental sciences, Planetary science

## Abstract

Microbial impacts on early carbonate diagenesis, particularly the formation of Mg-carbonates at low temperatures, have long eluded scientists. Our breakthrough laboratory experiments with two species of halophilic aerobic bacteria and marine carbonate grains reveal that these bacteria created a distinctive protodolomite (disordered dolomite) rim around the grains. Scanning Electron Microscopy (SEM) and X-ray Diffraction (XRD) confirmed the protodolomite formation, while solid-state nuclear magnetic resonance (NMR) revealed bacterial interactions with carboxylated organic matter, such as extracellular polymeric substances (EPS). We observed a significant carbon isotope fractionation (average δ^13^C = 11.3‰) and notable changes in Mg/Ca ratios throughout the experiments. Initial medium δ^13^C was − 18‰, sterile sediments were at 2‰ (n = 12), bacterial-altered sediments were − 6.8‰ (n = 12), and final medium δ^13^C was − 4.7‰. These results highlight the role of bacteria in driving organic carbon sequestration into Mg-rich carbonates and demonstrate the utility of NMR as a tool for detecting microbial biosignatures. This has significant implications for understanding carbonate diagenesis (dissolution and reprecipitation), climate science, and extraterrestrial research.

## Introduction

Carbonates serve as are valuable archives of Earth’s surface evolution^1–6^. However, during early diagenesis, these sediments undergo biogeochemical interactions with pore waters, leading to significant modifications in their primary chemical and isotopic compositions^7,8^. Early diagenesis is commonly the first step of altering primary metastable carbonate polymorphs such as aragonite (CaCO_3_), high Mg-calcite (HMC: > 4 molar% MgCO_3_; ([Ca,Mg)CO_3_] into the more thermodynamically stable phases such as low Mg-calcite (LMC: < 4 molar% MgCO_3_) and/or dolomite [CaMg(CO_3_)_2_], in the presence of seawater and related fluids^8–12^. The geochemical signatures of carbonate rocks, including isotopic and elemental compositions, have been extensively documented as proxies for quantifying the influence of early diagenesis^6,12–16^. This leads to enhanced accuracy in records reflecting the chemical and climatic history of the Earth, particularly regarding global carbon and oxygen cycles. Enhancing our comprehension of the dissolution–precipitation and mass-flux mechanisms linked with early diagenesis will bolster numerical and geochemical models depicting present and past interactions between Earth’s surface and fluids. As early diagenetic processes have been extensively investigated and documented to support environmental studies^12^, a more profound understanding is required regarding the specific mechanisms of mineralogical and geochemical alteration induced by microbial activity in shallow carbonate environments.

Indeed, the phenomenon of micro-bioerosion, and how microbes alter the grains morphologically, has been extensively examined over the past decades^17–20^. However, the contribution of microbes to geochemical alterations of grains during early marine diagenesis has been largely overlooked and remains poorly understood. Particularly, the biogeochemical pathways leading to the formation of Mg-rich carbonates under Earth’s surface conditions pose a significant enigma, prompting extensive research both in the field and laboratory settings. In recent years, there has been a notable increase in research endeavors aimed at addressing this knowledge gap^6,14–16,21–27^*.* Moreover, it is evident that in modern saline environments, microbes are involved in the precipitation of sedimentary carbonates^22,28–38^. A wide range of aerobic and anaerobic microorganisms such as methanogenic, heterotrophic and phototrophic bacteria are known to mediate the precipitation of carbonates^6,15,16,22–26,30–43^.

More recently, studies have emphasized the potential of aerobic biomineralization in the formation of Mg-rich carbonates and their implications towards natural environments^4–37^. Moderately halophilic aerobic bacteria (MHAB) are known to mediate the formation of Ca–Mg carbonates at Earth’s surface sedimentary conditions^22–24,32,34,37,42,43^. However, the precise geochemical interactions between MHAB and different types of carbonate sediments and their associated geochemical and morphological/textural alterations are unknown. Moreover, MHAB can thrive in a wide range of salinity variations and, hence, are good indicators, the influence of the ionic composition of the environment has on mineral precipitation induced by bacteria^22,43^. Therefore, these microbes are excellent candidates for controlled mineralization experiments in the laboratory, which can elucidate the mechanisms of natural Mg-rich carbonate formation in nature.

The aim of this study is to investigate the alteration of early carbonate grains induced by aerobic heterotrophic bacteria in order to understand the underlying alteration mechanisms. This study specifically focusses on two MHAB strains, *Virgibacillus marismortui* and *Halomonas meridiana*, which are commonly found in the microbial ecosystems of shallow carbonate environments^22,34,42,43^. The primary objective is to examine the interplay between these bacteria and the carbonate sediment, and to analyze the resulting alterations of sediment composition during laboratory culture experiments. The findings of this study offer valuable insights into the relationship between mineralogical, morphological and geochemical changes in carbonates during early carbonate deposition by MHAB, thereby enhancing our understanding of Ca–Mg carbonate formation in natural environments. This study introduces a novel multidisciplinary approach to investigating carbonate grain alteration mediated by microbial activity, utilizing elemental concentrations, stable isotope, electron microscopy and solid-state NMR analyses. By leveraging these techniques, critical insights will be provided into biogeochemical processes occurring within carbonate sediments.

## Results

### Mineralogical composition

Sediment recovered from *V. marismortui* culture experiment (Aa3V) is composed of Mg-rich carbonates (9% low Mg-calcite; 3% high Mg-calcite; 5% disordered dolomite or protodolomite with a d_104_ = 2.885 Å) and Ca-carbonate (82% aragonite), while sediment from control experiment (Aa1) is composed of aragonite (~ 97%), with minor amounts of low (2%) and high (1%) Mg-calcite. Sediment recovered from *V. marismortui* culture experiments (Rb3V) comprised 36% aragonite, 8% protodolomite (d_104_ = 2.927 Å), low (4%) and high (37%) Mg-calcite compared to sediment from control (Rb1), composed of 48% aragonite, low (2%) and high (50%) Mg-calcite. Sediment recovered from *H. meridiana* culture experiments (Rc3H) contained 86% aragonite, 5% low Mg-calcite and 7% disordered dolomite (d_104_ = 2.927 Å) whereas sediment from control experiment (Rc1) was composed of 94% aragonite, 2% low Mg-calcite (LMC) and 1% high Mg-calcite (HMC). Table 1, Fig. S3. pH in bacterial culture experiments increase from 7.2 to ~ 8.5–9. No changes in pH or Mg-rich carbonate precipitates were observed in the control experiments.

**Table 1:** Mineralogical composition (%) of the recovered substrate from bacterial culture and control (sterile substrate) experiments after 12 months of incubation at 30 °C under aerobic conditions.

	Aragonite	Low Mg-calcite (LMC)	LMC mol% Mg	High Mg-calcite (HMC)	HMC mol% Mg	Proto-dolomite	Proto-dolomite mol%Mg	Hydromagnesite
Aa1	97	2	1	1	12	0	0	0
Aa3V	83	9	4	3	19	5	40	0
Rb1	48	2	2	50	15	0	0	0
Rb3V	36	10	4	37	15	8	39	6
Rc1	94	2	2	1	18	0	0	0
Rc3H	86	7	4	0.1	18	7	36	2

Potential mineral phases and SI values for the initial conditions of the culture medium is reported in Table S1. According to these data, the medium investigated is undersaturated with respect to aragonite (SI = − 6.5), calcite (− 6.2), dolomite (SI = − 11.2) and hydromagnesite (SI = − 35.4) and saturated with respect to hydroxyapatite.

### Elemental concentration analyses

**Fig. 1:**
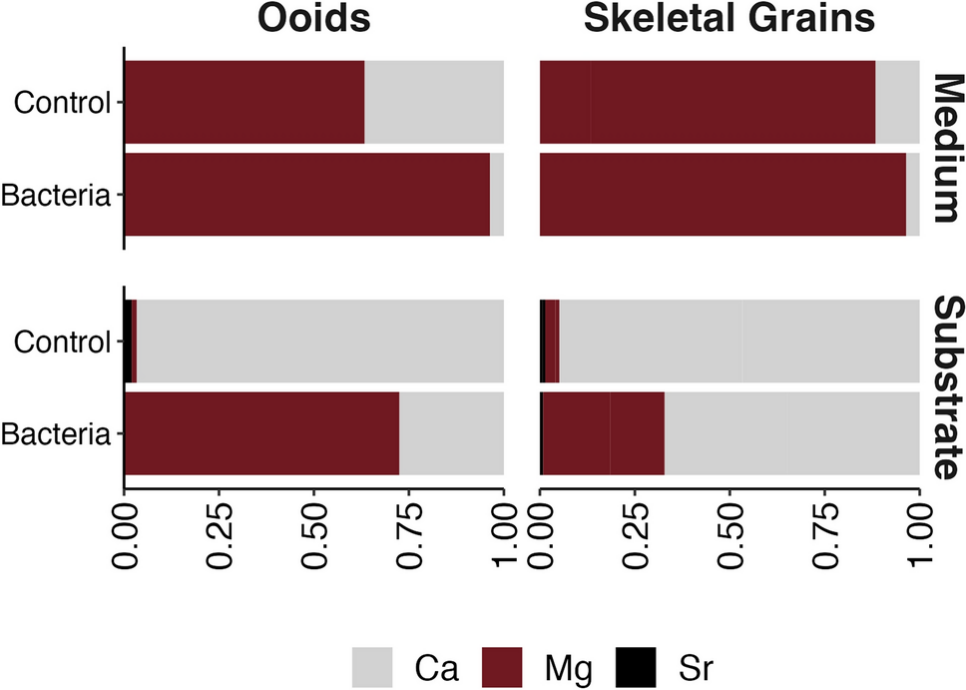
Box plot exhibiting the average variation in the concentration of major elements Ca, Mg, and Sr during the experiments in both sterile control and inoculated conditions for substrates and culture medium. A decrease in Ca and an increase in Mg is demonstrated from control (without bacteria) to inoculated substrates. Note that control medium is used as starting medium due the partial dissolution (increase in Mg, Ca and Sr) of the sediment during autoclaving.

Ca^2+^, Mg^2+^ and Sr^2+^ concentrations were measured in the solutions and of the substrates (Table 2, Fig. 1) recovered from bacterial culture and control experiments. The starting medium concentrations of Ca^2+^ (4.8 mM), Mg^2+^ (21.6 mM) and Sr^2+^ (0 mM) were the same for all experiments. However, due to autoclaving solutions of control experiments, recovered after 12 months, presented a significant increase in Mg^2+^ compared to the starting concentration (21.6 mM), with 80.3 mM for Aa1, 71.4 mM for Rb1 and 80.5 mM for Rc1). Ca^2+^ significantly increased, compared to the starting concentration (4.8 mM), in Aa1 (28.23 mM) and Rc1 (7.48 mM), and decreased in Rb1 to 2.25 mM. Sr was low in all measured control solutions (0.9 to 2.4) (Table 2). Hence, in this study, metal composition of final control solutions is used as the starting solutions because partial dissolution of the carbonate substrate occurred during the autoclaving process (at 120 °C for 20 min), leading to an increase in Mg, Ca, and Sr concentrations. The Ca and Mg final concentrations of the culture solutions, after mineral precipitation, showed a significant decrease. Ca^2+^ decreased from 28.2 (Aa1) to 0.5 in Aa3V, from 2.2 (Rb1) to 0.11(Rc1) mM in Rb3V, and from 7.5 to 0.5 mM in Rc3H experiments (Table 2). Mg^2+^ decreased from 80 to 23 mM in Aa3V, from 71.4 to 14.4 in Rb3V, and from 80.5 to 20.3 in Rc1 experiments. Sr^2+^ also decreased from 2.4 to 0.06 in Aa3V, from 0.13 to 0.02 in Rb3V, and from 0.91 to 0.04 in Rc3H experiments. See Table 2.

**Table 2:** Geochemical (elemental concentrations, Mg/Ca, C and O isotopes) composition of the final (after sediment substrate recovery) solutions from both bacterial culture and control experiments. Culture experiments were aerobically incubated at 30 °C; the starting pH was 7.2, and the final pH was ~ 8.5–9. The concentrations of Ca and Mg are in mmol (solutions) and wt% (substrates); Sr is in ppm. Values of isotopic compositions are in permille (‰) vs. VPDB for carbon and ‰ vs VSMOW for oxygen isotopes. Note that the final control solutions are used as the starting solutions because partial dissolution of the carbonate substrate occurred during the autoclaving process (at 120 °C for 20 min), leading to an increase in Mg, Ca, and Sr concentrations.

	Final control solution (starting culture solution)								Final culture solution (after sediment substrate recovery)								
	Ca	Mg	Sr	Mg:Ca	δ^13^C	Cstd	δ^18^Ο	Ostd		Ca	Mg	Sr	Mg:Ca	δ^13^C	Cstd	δ^18^Ο	Ostd
*Aa1*	28.2	80.3	2.4	22.8	–	–	–	–	*Aa3V*	0.5	23	0.06	43.1	− 4.8	0.15	− 6	0.03
*Rb1*	2.2	71.4	0.13	31.7	–	–	− 1.3	0.07	*Rb3V*	0.1	14.4	0.02	13.7	− 3.7	0.12	–	–
*Rc1*	7.5	80.5	0.91	20.8	–	–	− 0.4	0.03	*Rc3H*	0.5	20.3	0.04	44.4	− 5.6	0.01	–	–

	Sterile substrate (Control) (starting substrate)								Sediment substrate recovered from cultures								
	Ca	Mg	Sr	Mg:Ca	Avg. δ^13^C	Cstd	Avg. δ^18^O	Ostd		Ca	Mg	Sr	Mg:Ca	Avg. δ^13^C	Cstd	Avg. δ^18^O	Ostd
*Aa1*	35.8	0.5	7495	0.02	4.0 (n = 5)	0.2	0.2 (n = 5)	0.1	*Aa3V*	7.8	20.5	1355	4.34	− 9.5 (n = 4)	0.0	− 5.1 (n = 4)	0.1
*Rb1*	34.4	1.9	4833	0.09	2.2 (n = 3)	0.1	− 1.5 (n = 3)	0.1	*Rb3V*	20.4	11.2	1856	0.9	− 6.1 (n = 4)	0.1	− 3.7 (n = 4)	0.1
*Rc1*	33.3	0.7	5345	0.04	− 0.1 (n = 4)	0.0	− 0.9 (n = 4)	0.0	*Rc3H*	21.9	9	3535	0.68	− 4.7 (n = 5)	0.1	− 2.6 (n = 5)	0.1

Sediments recovered from MHAB experiments show an increase in Mg^2+^ and a decrease in Ca^2+^ and Sr^2+^ concentrations (Table 2; Fig. 1), indicating evolved dissolution and reprecipitation (recrystallization) processes during the incubation period of these experiments. In Aa sediment, an increase of Mg^2+^ from 0.5 wt% (Aa1, starting) to 20.5 wt% (Aa3V, final) occurred together with a decrease in Ca^2+^ from 35.8 wt% (Aa1) to 7.8 wt% (Aa3V) and for Sr^2+^ from 7495 ppm (Aa1) to 135.5 ppm (Aa3V). In Rb sediment, Mg^2+^ concentration increased from 1.9% (Rb1, starting) to 11.2 wt% (Rb3V, final), Ca^2+^ decreased from 34.4 wt% (Rb1) to 20.4 wt% (Rb3V) and Sr^2+^ diminished from 4833 (Rb1) to 1856 ppm (Rb3V). In Rc sediment, Mg^2+^ increased from 0.7 (Rc1, starting) to 9.0 wt% (Rc3H, final), Ca^2+^ declined from 33.3% (Rc1) to 21. 9 wt% (Rc3H) and Sr^2+^ changed from 5346 ppm (Rc1) to 3535 ppm (Rc3H).

### Carbon and oxygen stable isotope analyses

δ^13^C and δ^18^O values of the starting solution are − 18.1‰ ± 0.52 and − 6.5 ± 0.02‰, respectively. δ^13^C and δ^18^O values of the final solutions range from − 5.6 to − 3.7‰. and from − 1.3 to − 0.4‰, respectively (see Table 2).

δ^13^C and δ^18^O of the sediments were measured multiple times (3–6 times per sample; Table S2) for all bulk samples (Table 2). The sediments recovered from control experiments showed: Aa1 with δ^13^C values from 3.8 to 4‰ (average 4‰; n = 5) and δ^18^O values between 0 and 0.3‰ (average 0.2‰; n = 5); Rb1 contained δ^13^C values of 1.9–2.6‰ (average 2.2‰; n = 3) and δ^18^O values of − 1.8 to − 1.1‰ (average − 1.5%; n = 3); δ^13^C values in Rc1 ranged from − 1.5 to 1.1‰ (average − 0.1‰; n = 4) and δ^18^O values from − 1.8 to − 0.3‰ (average − 0.9‰; n = 4). See Table 2. All the sediments recovered from bacterial culture experiments showed a significant enrichment in the lighter isotopes ^12^C and ^16^O. Aa3V sediments disclosed δ^13^C values from − 11.5 to − 8.6‰ (average − 9.5‰; n = 4) and δ^18^O values from − 5.6 to − 4.9‰ (average − 5.1‰; n = 4). In Rb3V sediments the δ^13^C values varied from − 10.18 to − 2.71‰ (average − 6.1‰; n = 4), and the δ^18^O values from − 4.4 to − 2.7‰ (average − 3.7‰; n = 4). Rc3H sediments showed δ^13^C values ranging from − 6.2 to − 3.6‰ (average − 4.7‰; n = 5) and δ^18^O values between − 2.8 and − 2.3‰ (average − 2.1‰; n = 5).

### ^13^C cross-polarization (CP) and direct polarization (DP) magic angle spin (MAS) solid state nuclear magnetic resonance (NMR) analysis

**Fig. 2:**
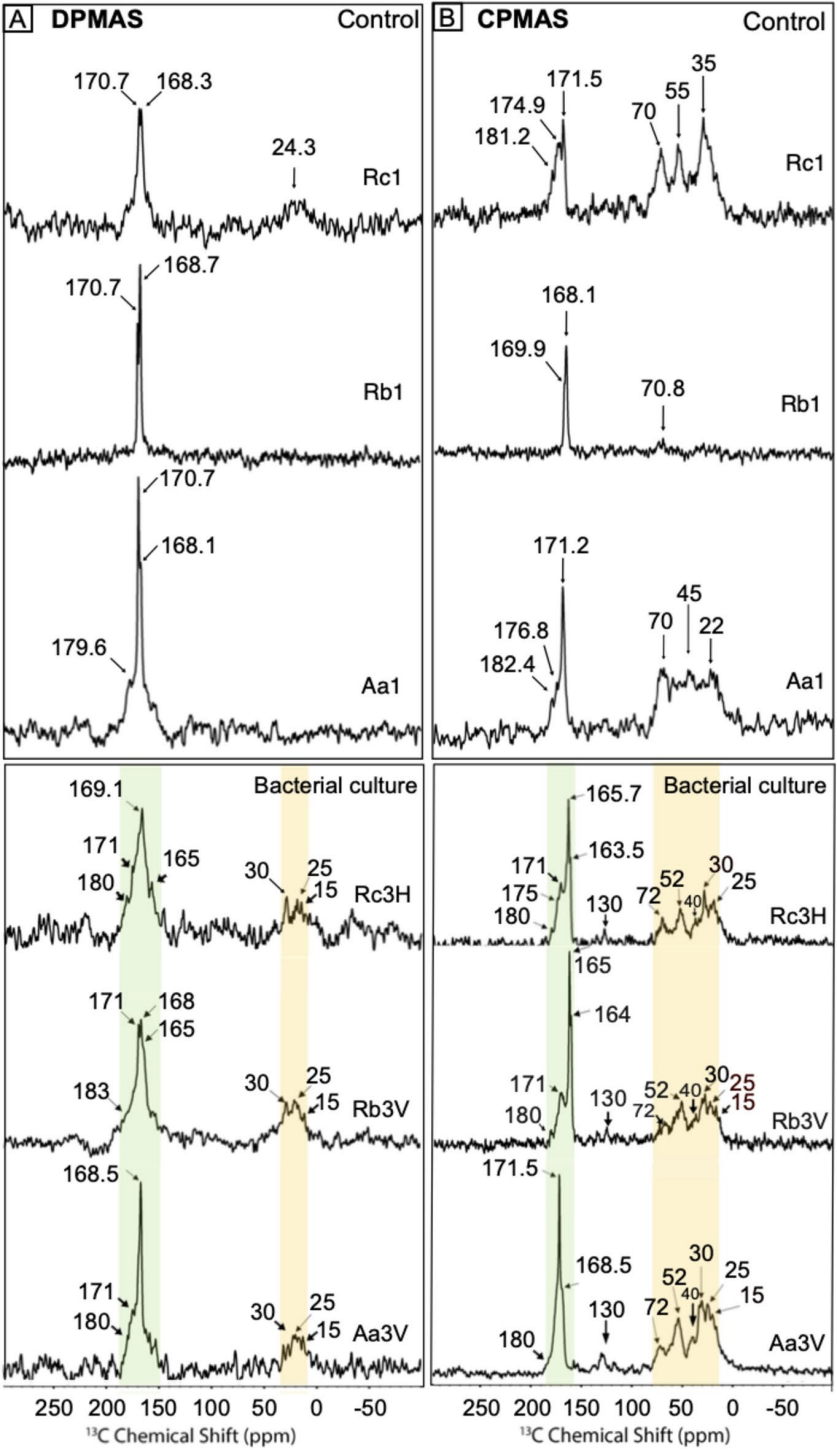
(**A**) ^13^C Direct-Polarization magic angle spinning (DPMAS) NMR spectra of sediments recovered from control (Rc1, Rb1, Aa1) and *H. meridiana* (Rc3H) and *V. marismortui* (Rb3V, Aa3V) culture experiments. Yellow and green shaded areas highlight the regions with increased ranges of ^13^C chemical shifts associated with bioorganic content. (**B**) ^13^C Cross-Polarization magic angle spinning (CPMAS) NMR spectra of sediments recovered from control (Aa1, Rb1, Rc1) and *V. marismortui* (Aa3V, Rb3V) and *H. meridiana* (Rc3H) culture experiments. Yellow and green shaded areas highlight the regions with increased ranges of ^13^C chemical shifts associated with bioorganic content.

Generally, CPMAS-NMR is more sensitive to even smaller chemical shifts, while DPMAS-NMR is more quantitatively indicative of the presence of a specific peak or signature. The ^13^C DPMAS-NMR spectra of the control sediments (Aa1, Rb1, Rc1) shows a predominant peak at δ_C_ = 170.7 ppm and a shoulder peak at δ_C_ = 168.1–168.7 ppm. No major peaks associated with organic carbon are present in the ^13^C DPMAS-NMR spectra of any of the control samples. All sediments recovered from MHAB experiments (Aa3V, Rb3V and Rc3H) display wide ^13^C DPMAS-NMR peaks in the δ_C_ range of 160–190 ppm. Aa3V, Rb3V and Rc3H sediments also exhibited distinct broad peaks of ^13^C DPMAS-NMR in the δ_C_ 15–30 ppm range which are associated with organic carbon (Fig. 2A).

The cross-polarization in the ^13^C{^1^H} CPMAS-NMR spectra enhance the signal of carbon atoms with protons in the vicinity. Therefore, we do not expect to observe any major peaks associated with proton-free phases such as calcite and aragonite in the ^13^C CPMAS spectra^44–47^. ^13^C{^1^H} CPMAS-NMR hence allows us to distinguish the signal of Mg-rich carbonate peaks with structurally incorporated water in the various carbonate phases as well as organic carbon associated with bio-organic content. The ^13^C{^1^H} CPMAS-NMR spectra of control sediments Aa1 and Rc1 showed a sharp peak around δ_C_ = 171, while Rb1 around δ_C_ = 168 ppm reflecting the higher content of magnesium calcite phases compared to Aa1 and Rc1. Aa1 and Rc1 sediment also showed minor ^13^C{^1^H} CPMAS-NMR peaks in the ranges δ_C_ = 182–176 ppm and δ_C_ = 70–55 and 35–22 ppm, which is attributed to the organic carbon originating from the experimental solution. In contrast to the control experiments, sediments from MHAB culture experiments displayed a broaden ^13^C{^1^H} CPMAS-NMR chemical shift range between δ_C_ = 170 ppm and 168.5 ppm for Aa3V; and between δ_C_ = 180 ppm and 163.5 ppm for Rb3Vand Rc3H (see Fig. 2B). All sediments recovered from culture experiments showed minor ^13^C{^1^H} CPMAS-NMR peaks at δ_C_ = 130 ppm and in the ranges δ_C_ = 70–52 and 40–25 ppm.

### Scanning electron microscopy (SEM) analysis

**Fig. 3:**
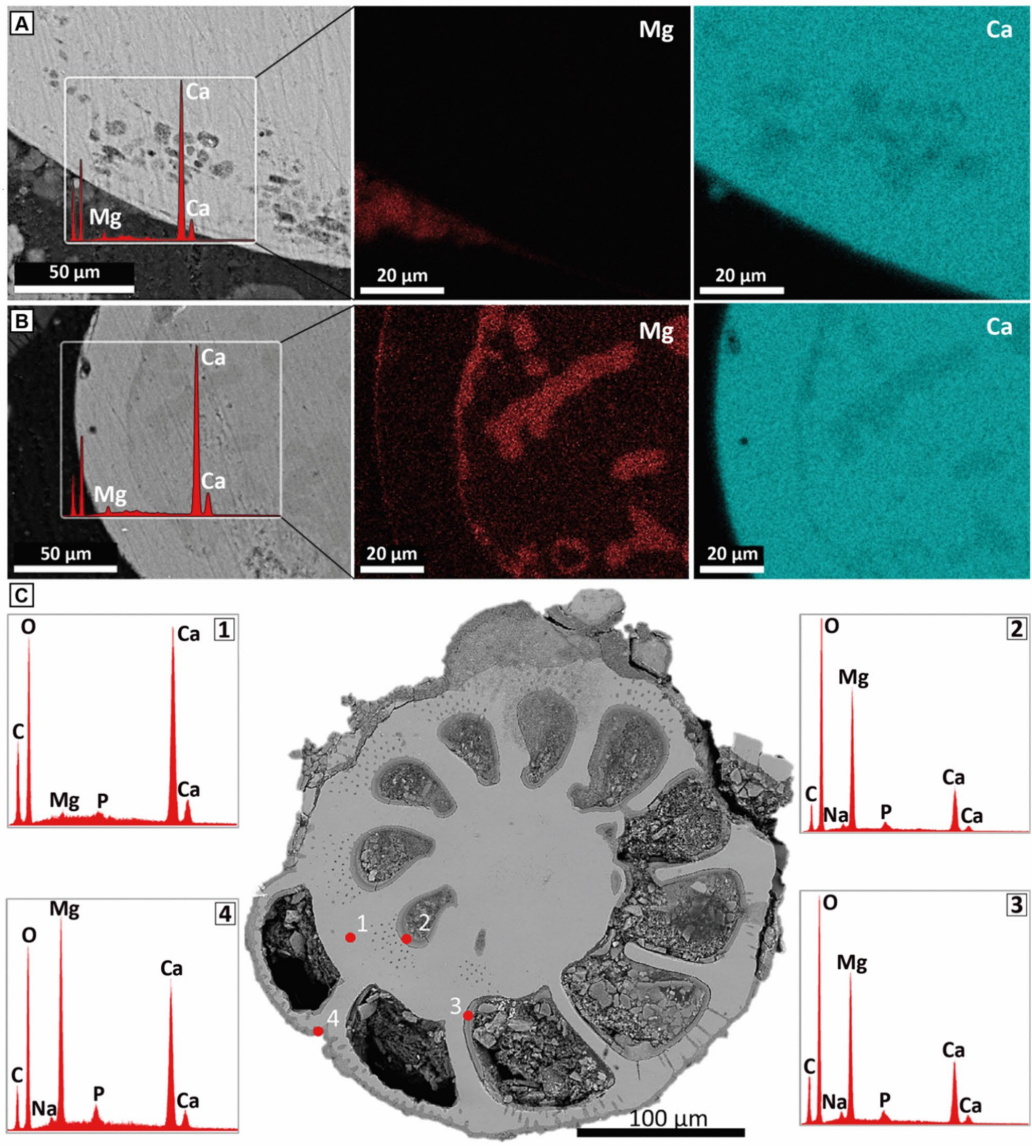
SEM analysis of Arabian Gulf sediments from (**A**) non-inoculated substrate (Aa1) and (**B**) inoculation experiments (Aa3V). The insets show the areas where EDX analysis was performed and the respective Mg and Ca distribution maps on the right. (**C**) A representative SEM back-scatter image of foraminifera from the Red Sea sediments recovered from MHAB culture experiments (Rb3V). Points 1, 2, 3, and 4 indicate where EDX analyses were performed. The inner (2) and outer (3) chamber walls of the foraminifera, as well as the outer rim (4), are enriched by Mg-rich carbonate precipitates, with significantly higher Mg mol% compared to the skeletal matrix (1).

SEM images of the autoclaved sediments (Aa, Rb and Rc) prior to negative control and inoculated experiments confirmed variable degrees of micritization of the carbonate grains, ranging from partially to completely filled microborings (Fig. 3). However, we do not observe any ring-forming precipitation, a clear rim around the carbonate grains, or any alteration of grain composition (Fig. 3A), unlike the precipitates recovered from bacterial cultures (Fig. 3B). Rb1 and Rc1 grains showed higher Mg wt% levels compared to Aa1, reflecting the originally high Mg-calcite composition in these sediments. In sharp contrast, all sediments inoculated with MHAB are associated with variable levels of Mg-enrichment around and within the carbonate grains, as well as on the pore walls (Fig. 3B,C; Fig. S3). The Aa3V carbonate grains show Mg-rich rims and an increase of mol% Mg in the previously micritized grain volume (Fig. 3B,C). Pore-filling Mg-rich carbonate precipitation is more abundant in Rc3H compared to Rb3V. The mineral composition and microtextures of the mineral precipitates recovered from experiments inoculated with *V. marismortui* (Aa3V, Rb3V) and *H. meridiana* (Rc3H) contain Mg-rich carbonates (protodolomite and hydromagnesite) (Fig. S3).

## Discussion

### Carbonate alteration by heterotrophic aerobic bacteria

Mineralogical and geochemical data indicate that all the sediments recovered from bacterial culture experiments are enriched in Mg-rich carbonates compared to the sediment recovered from control experiments. This alteration of the sediment grains is mainly attributed to the overall changes in the percentages of aragonite, LMC, HMC, and protodolomite, as well as the increase in their respective Mg mol% (Table 1). Carbonate crystallinity is represented by sharp peaks in the XRD, whereas the widespread peaks are due to the incorporation of Mg^2+^ in the crystal lattice of the carbonates and mixture of amorphous, organic compounds and crystalline mineral precipitates (Fig. S2). The d_104_ of the protodolomite precipitates is 2.885 Å for Aa3V and 2.927 Å for Rb3V and Rc3H, which are considered disordered dolomites compared to ordered dolomite, where d_104_ = 2.897 Å. The d_104_ of protodolomite precipitates from MHAB falls within the range of previously reported disordered dolomite from solutions containing ethanol and calcite seeds^44^ (d_104_ = 2.916, 2.918, 2.936, 2.940) and from bacterial experiments^45^ (d_104_ = 2.907, 2.904, 2.916). As XRD is mainly indicative of the overall shape of the molecule and the crystal lattice structures, NMR is sensitive to atomic detail and molecular bonds^46–50^. NMR can, thus, improve the accuracy of X-ray structures at atomic resolution. In the NMR data, the broader peaks are indicative of the presence of ^13^C in mineral phases in environments enriched in organic components.

**Fig. 4:**
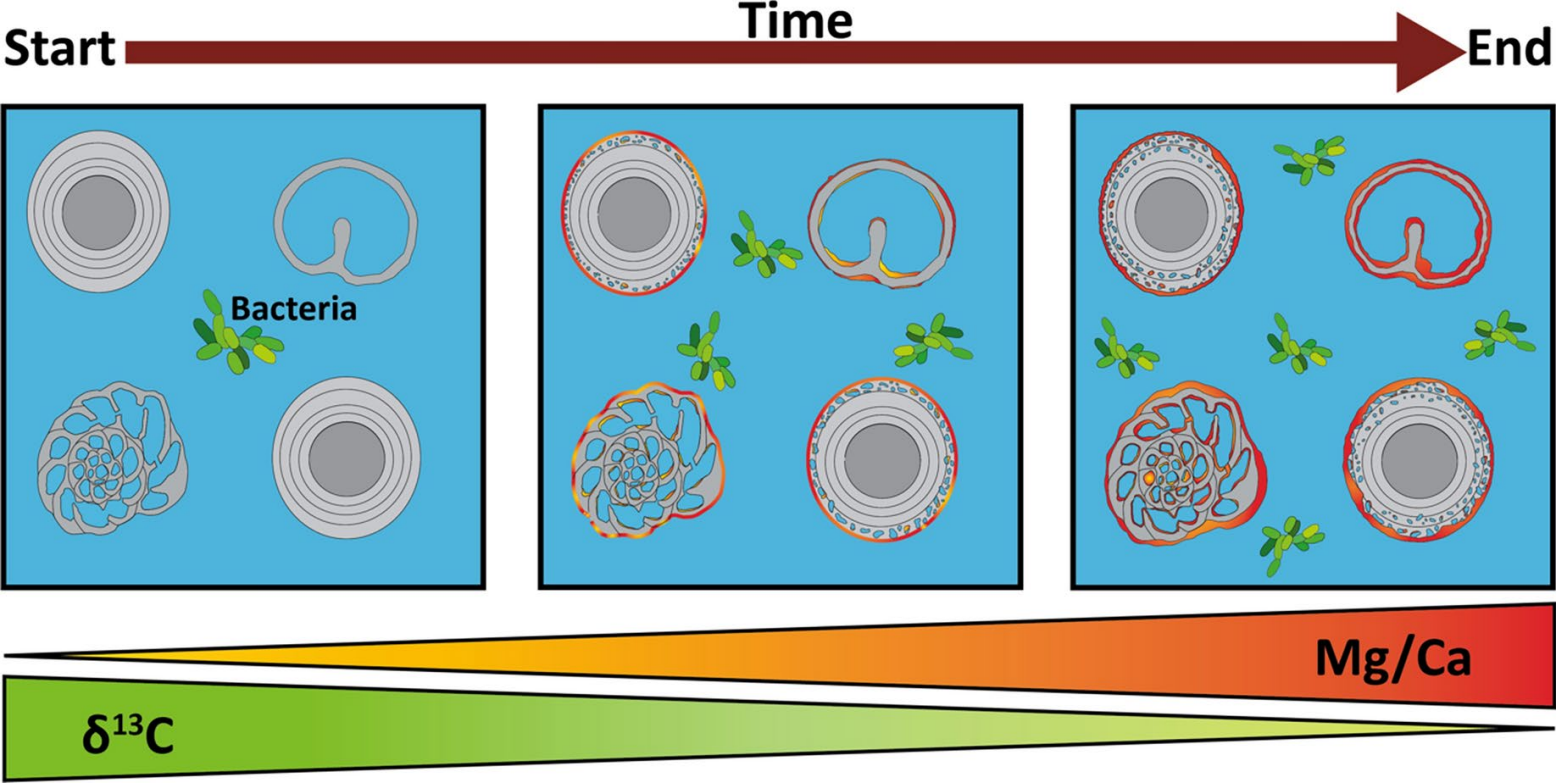
Schematic representing the three modes of alteration observed in the sediment grains when exposed to bacteria (*V*. *marismortui* and *H*. *meridiana*) for an extended period.

Three modes of alterations in the MHAB inoculated substrates were observed (Fig. 4): (i) precipitation of Mg-rich carbonates on the grain surfaces and pore walls manifested as Mg-rich rims (Fig. 3B,C); (ii) precipitation of Mg-rich carbonates in the intragranular pore space (Fig. 3B) and (iii) Mg-enrichment in previously micritized pore space (Fig. 3B,C). The alteration of grains is sensitive to mineral-microbe interactions, causing dissolution and precipitation within the grains changing the mineralogical and geochemical composition of the grains.

In control sediments (Aa1, Rb1, Rc1; Fig. 2A), ^13^C DPMAS predominant peaks at δ_C_ = 168.1–168.7 ppm, confirm the local structural environment of carbon in aragonite and Mg-calcite^46–50^. A shift from calcite or aragonite peaks towards lower δ_C_ values is attributed to the presence of Mg^2+^ (Fig. 2A), lower δ_C_ values in Rb1 (168.7 ppm, major peak) and Rc1 (168.3 ppm, major peak) reflect a higher Mg content than in the Aa1 sediment (major peak at δ_C_ = 170.7 ppm). In sediments recovered from MHAB culture experiments (Aa3V, Rb3V, Rc3H; Fig. 2B), ^13^C DPMAS broad peaks at δ_C_ = 165–171 ppm correspond to a combination of increased levels of Mg and organic carbon (carboxylate groups)^49–55^; whereas small broad peaks at δ_C_ = 30–15 ppm indicate significantly higher levels of organic carbon^53–56^ than in the control sediments. The ^13^C {^1^H} CPMAS of Aa1 and Rc1control sediments showed minor peaks for δ_C_ = 181–182 ppm and a broad range between δ_C_ = 70–55 and 45–22 ppm, which can be attributed to the organic carbon originating from the organic compounds used in the experimental solution. In contrast to the control sediments, ^13^C {^1^H} CPMAS of Rb3V and Rc3H sediments recovered from bacterial cultures (Fig. 2B) showed a sharp peak at δ_C_ ~ 165.3 ppm with shoulder peaks at δ_C_ = 163.5–163.8 ppm, indicative of Mg-rich carbonates with structurally incorporated water, including hydromagnesite and dypingite^50–53^, reflecting the content of water-containing Mg-carbonate phases (hydromagnesite) compared to Aa3V where hydromagnesite is absent. All sediments recovered from bacterial cultures (Aa3V, Rb3V, and Rc3H) displayed distinct shoulder peaks at δ_C_ = 171 ppm and 168.5 ppm which may indicate water content in the protodolomite as previously has been reported^57,58^. Aa3V, Rb3V, and Rc3H also exhibited a broadened range of minor peaks at δ_C_ = 130 ppm and a broad range between δ_C_ = 72–52 ppm and 40–15 ppm, indicating the presence of organic carbon, particularly amide and carboxylate carbons from bio-organic (bacterial) material^51–56^. Additionally, characteristic signals of amine groups associated with peptides were also observed^51–54^. The peaks identified in the spectra correspond to various carbon types: methyl C (δ_C_ = 15 and 25 ppm) and methylene C (δ_C_ = 30 ppm)^59,60^, methine C (δ_C_ = 40 ppm)^60^, and aliphatic Cα (δ_C_ = 52 ppm)^59^. Polysaccharides show peaks between δ_C_ = 72 ppm (C2-C6) and 130 ppm for bridging aromatics^59,60^. Methylene groups (δ_C_ = 25–35 ppm) relate to bacterial membrane lipids, while O/N-aliphatic C (δ_C_ = 40–70 ppm) indicates amino acids in peptides and polysaccharides^61,62^. EPS protein structures are composed of peptides and amides, whereas polysaccharide structures primarily consist of O-alkyl, ring, and anomeric carbon^61,62^. The presence of amide and carboxylate groups is linked to bacterial protodolomite precipitation in MHAB cultures, aligning with previous findings that report organic matter in bacterial protodolomite^45^.

**Fig. 5:**
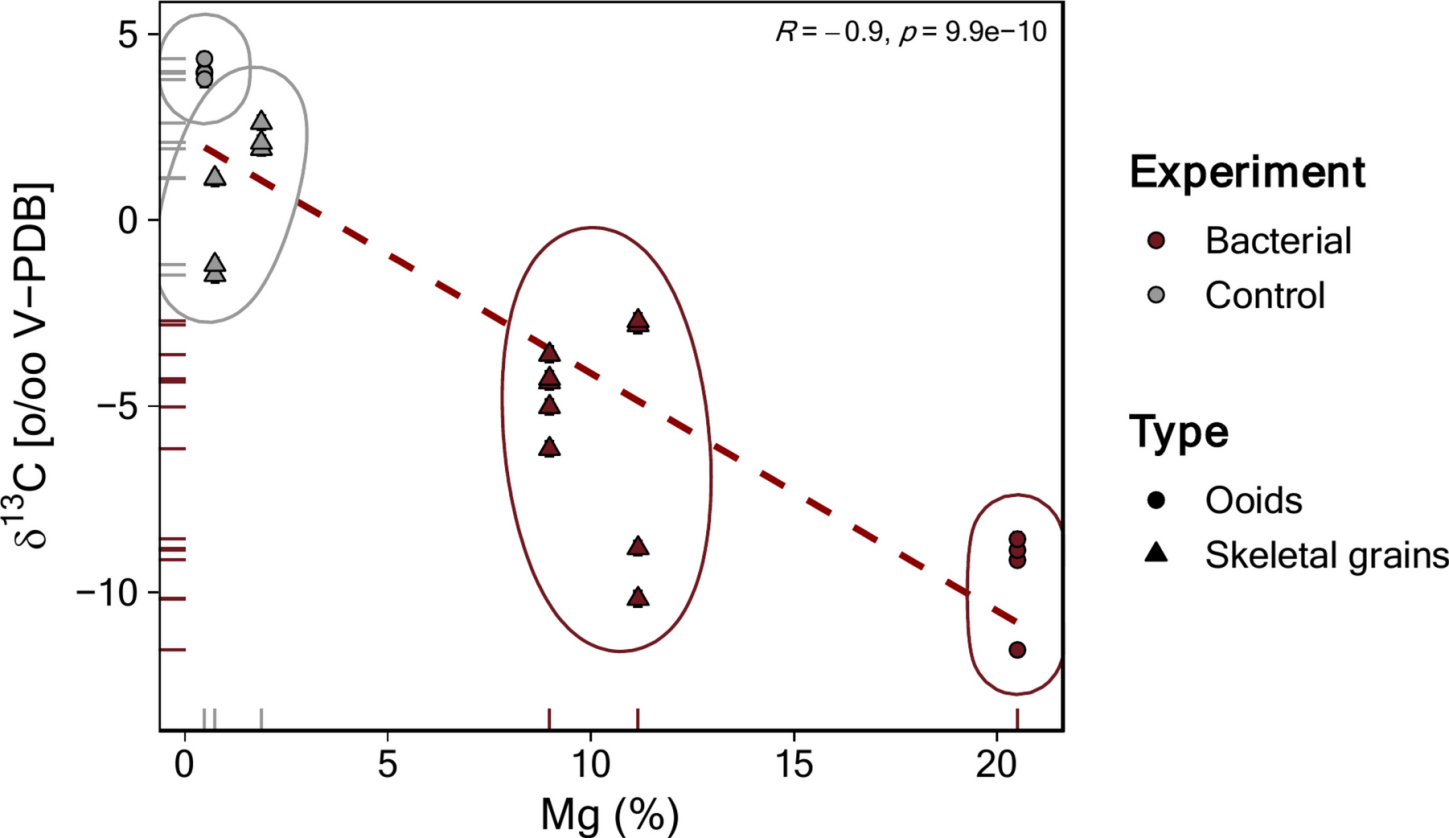
Stable carbon isotopic composition (^13^C) vs. Mg concentration of the experimental bulk carbonate sediments. A positive relationship exists between the more negative δ^13^C values and increased Mg^2+^ concentration in bulk sediments. Samples show clusters by substrate, indicating the influential role of microbial activity in altering the substrate and incorporating Mg.

SEM results also confirm the Mg-enrichment in the sediments from the bacterial cultures. Not only within the carbonate grains, forming a rim, but in some cases also within the grain pore-space (Fig. 3B,C). All natural sediments were already micritized before the experiments, and the mineralogy of the pore-filling micrite in these sediments is predominantly aragonite. However, after being exposed to MHAB, the mineralogy of the pore-filling changed to predominantly Mg-rich carbonates (HMC, protodolomite). Hence, bacterial activity enhanced the precipitation of Mg-rich carbonates. The accretion of thin Mg-rich carbonate layers in the grains, distinct biogenic signatures in NMR, and the ^13^C-depleted isotopic composition indicate the bacterial role in this alteration process. The carbon of biogenic and authigenic carbonates is derived from CO_3_^2−^ ions dissolved in the precipitating solution. Likely, the oxygen isotopic composition of carbonates is a function of the solution and the temperature in which the carbonate is precipitating^63–66^. Since, dissolved inorganic carbon (DIC) in the experiments is expected to be in equilibrium with the atmosphere and the temperature remained the same during the entire course of the experiments, the observed offset in δ^13^C (− 8.8‰) and δ^18^O (− 3.05‰) between the control and bacterial culture experiments must be caused by the bacterial fractionation during the precipitation of new Mg-rich carbonate minerals (HMC, protodolomite). Moreover, the strong negative correlation (R = − 0.9) between δ^13^C and Mg%, shows that the ^13^C-depleted samples are more Mg-enriched (Fig. 5). These new Mg-rich carbonate precipitates are enriched in ^12^C derived from the decomposition of organic matter during respiration^24^. Our findings support that microbial activity promotes and facilitates the incorporation of metals, in this case Mg^2+^, into the lattice of carbonate crystals at low temperatures (< 50 °C). This process also modifies pre-existing carbonate grains with subsequent partial dissolution-reprecipitation, and initiate dolomitization reactions during early diagenesis. *H. meridiana and V. marismortui* can replace Ca^2^⁺ with Mg^2^⁺, thereby inducing carbonate precipitation. Our results show a decrease in Ca^2^⁺ and an increase in Mg^2^⁺ concentrations in all experimental setups compared to the control substrates. This observation indicates that microorganisms can actively alter their microenvironment, forming localized microzones of dissolution and reprecipitation that drive and facilitate these chemical transformations.

The bacterial alteration observed in the sediment grains is more apparent in the aragonite-rich sediment Aa1, which contains less Mg-calcite compared to both Rb1 and Rc1; latter contains more Mg-calcite. Sediment recovered from bacterial cultures displayed a significant decrease in aragonite concurrent with the formation of new precipitates, including protodolomite (up to 40 mol% Mg) and hydromagnesite (Table 1, Fig. S2). This is consistent with the increase in the Mg/Ca ratio of the bulk sediment from the MHAB experiments (Table 2, Figs. 4, 5).

Bacterial metabolism affects the magnitude of isotope fractionation, indicating an exchange of C isotopes between carbonate minerals and the precipitating solution (Table 2). The corresponding C isotope compositions showed a fractionation sequence from the isotopically lightest bulk solution (δ^13^C = − 18.1‰) to the isotopically light bulk sediment recovered from bacterial cultures (δ^13^C_average_: Aa3V = − 9.5‰ n = 4, Rb3V = − 6.1‰ n = 4, Rc3H = − 4.7‰ n = 5; Table 2 and Table S1). The recovered sediments from bacterial cultures showed an ^12^C-enrichment of − 13‰ (Aa3V), − 8.1‰ (Rb3V) and − 3.6‰ (Rc3H), compared to the heavier δ^13^C starting sediment (control: Aa1 = 4‰; Rb1 = 2.2‰, Rc1 = − 0.1‰). At the same time the Mg concentrations in the solutions increased from 21 to 43.1 mM (Aa3V), 31.7 mM (Rb1) and 44.4 mM (Rc3H). This increase further demonstrates that MHAB can have a significant role during early diagenetical processes like micritization, dissolution and (re)precipitation of carbonates (e.g., dolomitization). Recent studies demonstrated that under aerobic conditions at the sediment–water interface, a decrease of δ^13^C already occurs in carbonate sediments, which continues to decrease with depth^12,60^.

### Microbial processes in carbonate precipitation and sediment alteration

Previous studies of *H. meridiana* and *V. marismortui* have found one of the top metabolic pathways of these microbes to be amino acid and carbohydrate metabolism^22–24^. They both are known to cause an increase of pH in the solution from a starting value of 7 up to 8.5–9^22–24,34,42^. They produce ammonia (NH_3_) through the oxidative deamination of amino acids, leading to an increase in the overall pH and alkalinity within the microenvironment surrounding the cells, resulting in carbonate precipitation both on the cells and in their secreted extracellular polymeric substances (EPS)^22–24,29,34^. MHAB also produces CO_2_, which at higher pH levels dissolves and converts into HCO_3_^−^ or CO_3_^2–24^. The decrease in δ^18^O in the bacteria inoculated sediments (Aa3V, Rb3V, Rc3H; Table 2) suggests kinetic fractionation of δ^18^O due to hydration or hydroxylation of CO_2_^65^.

The bacterial degradation of organic matter releases the lighter ^12^C isotope, which also contributes to the formation of the CO_3_^2−^ ions^22–24^, which is reflected in the δ^13^C displaying more negative values of up to − 11.5‰ in the experimentally altered sediments (Fig. 5; Table S2)^24^. In the presence of Ca^2+^ and Mg^2+^ ions, the solution can reach supersaturation with respect to Mg-rich carbonates^22–24^, a process facilitated by functional groups (e.g., carboxyl groups) in EPS or cell walls that adsorb Mg^2+^ ions. This adsorption not only creates a localized supersaturated environment^22–24^ but also lowers the activation energy required for crystal growth, for instance, by dehydrating Mg^2+^ ions^67,68^. This combination of increased pH, carbonate ions, and dehydration of Mg^2+^ promotes carbonate precipitation and replacement of Ca^2+^ by Mg^2+^ (ion exchange mechanism) on the grain surface-pore water interfaces, within and around the sediment grains, following kinetic pathways that can leverage the alkalinity engine^22–24,66^. Such precipitation mechanism of Mg-rich carbonate mineralization, in the studied carbonate sediments inoculated with bacteria, is evident from the mineralogical, geochemical, spectroscopic and microscopic analyses reported in the present study.

The presence of *V. marismortui* and *H. meridiana* can alter the solution/interface chemistry due to the presence of simple carboxylic acids and acidic proteins in their cell walls and EPS. These biomolecules can regulate the Mg composition in the precipitated carbonates with up to 50 mol% Mg, as in HMC and protodolomite, potentially involving an intermittent Mg-ACC phase^69^. The overall increase in alkaline pH, previously expounded, destabilizes the functional groups of the EPS and causes deprotonation. This negative charge then attracts the cations (Mg^2+^, Ca^2+^) to bind with the interface and initiate nucleation of carbonate minerals. It has been observed that formation of dolomite nanoglobules (< 200 nm) associated with organic films (EPS) and bacterial cells, arranged in chains or clusters resembling the cells, aggregate into spherical or ovoidal shapes^23^. Based on AFM and TEM analysis^23^, the cell sizes of *V. marismortui* and *H. meridiana* suggest their ability to colonize both macro- and micropores in sediment grains, facilitating the formation of EPS within pores that are large enough to accommodate them, in addition to the more accessible surfaces around and within the sediment grains. The subsequent microbial micritic cementation of these grains enhances their stability, strengthening marine sediment similar to other types like soil or sand. This process also holds promise for protecting and restoring stone and concrete structures. Microbial cements mediated by bacteria offers innovative applications, including reinforcing natural and artificial marine structures and preventing coastal erosion^70^.

^13^C NMR data (Fig. 2) provided clear evidence of the presence of organic material in association with the new Mg-rich carbonate precipitated in the bacterial culture experiments. This supports the previously suggested mechanism, that the precipitation of Mg-rich carbonates is initiated by the accumulation of Ca^2+^ and Mg^2+^ ions on bacterial the cell walls/envelopes and EPS^6,22–24^. Mineralization of bacterial cells and EPS could have also contributed to the organic-rich signal observed in the NMR data. Moreover, depletion of δ^13^C is generally attributed to metabolic respiration by heterotrophic and methanogenic bacteria processes^6,15,16,28,31,35,60,71^. Therefore, authigenic carbonates in shallow marine environments such as micrite and cement generated during micritization generally show a bulk negative δ^13^C signature (approximately − 10 to − 1‰)^72^, as observed in our results, being up to − 11‰ when bacterially altered into a Mg-rich micrite, dolomicrite. The ^12^C- and Mg-enrichment after bacterial interactions with the grains is also supported by solid-state NMR and SEM analyses which indicate that the altered carbonate grains are in close association with organic compounds such carboxylated surfaces (bacterial cells, EPS). We expect the mechanisms driving the alteration of sediment grains by MHAB to occur through two main modes: (i) microbial influence on the physicochemical conditions of the solution (Mg/Ca, alkalinity, pH), resulting in dissolution-reprecipitation reactions at the solution-grain interfaces, and (ii) microbial-induced mineral precipitation associated with bacterial cells and EPS.

### Microbial carbonates in natural environments

The aragonite-calcite precipitation was mainly dictated by the seawater chemistry, which can indirectly influence skeletal mineralization by affecting the physiological costs of biomineralization^73–75^. However, it is well known that in shallow marine carbonate factories, microbes cause local changes in carbonate precipitation kinetics that can impact the formation pathways of carbonate sediments^6,38,76,77^. *V. marismortui* and *H. meridiana* are both known to tolerate high sulphate concentrations^22^ and so, could be less sensitive to seawater chemistry. *Virgibacillus* and *Halomonas* genus are well-known to alter pH (from 7.0 to 9) in laboratory culture experiments^22–24,34,42^. Therefore, the mechanisms of Mg-enrichment of carbonate grains observed in this study are, hence, relevant to natural shallow environments populated by these microbes.

XRD and SEM–EDS results show that in the bacterially altered sediments, a large fraction of aragonite was altered to Mg-calcite and protodolomite (Table 1). These minerals also constitute a substantial proportion of the mineralogy found in lime muds of carbonate reservoirs and significantly contribute to the development of microporosity^78^. In the Jurassic and Cretaceous carbonate formations in the Middle East, the mineralogy of precursor sediments (aragonite, HMC) was altered almost entirely to LMC and dolomite^79,80^. Our experiments demonstrate that heterotrophic bacteria can facilitate the alteration of carbonate minerals and initiate dolomitization through dissolution and precipitation reactions during early diagenesis. This study demonstrates that sediment alteration by aerobic heterotrophic bacteria involves changes in the physicochemical conditions of the surrounding environment (e.g., Mg/Ca ratio, CO_3_^2−^, pH), leading to dissolution/precipitation reactions. Therefore, we propose that MHAB not only play a crucial role in the formation of minerals in natural environments as previously suggested, but also could be potent mediators of grain alteration and, thus, could play an important role in the evolution of micro- and macro-porosity during early and shallow burial processes. Thus, bacterial micritization might have played a crucial role in enhancing and preserving the primary porosity in carbonates.

### Ecological and societal implications

Our findings impact various environmental and societal issues, including:

(i) Ecosystem Dynamics. The comprehension of the microbiological mechanisms underlying carbonate alteration advances our understanding of the dynamics of shallow-marine ecosystems and will help to comprehend the formation of submarine hardgrounds, stabilization of shallow-water sediments and other sediment capture processes.
(ii) Climate Change. Research on the evolution of porosity and micrite production sheds light on the effect that microbial activity might have on the carbon cycle and climate feedback mechanisms as these processes steer the dissolution and formation of carbonate sediments and cements.
(iii) Resource Management. Strategies for managing groundwater aquifers, carbon capture reservoirs, and hydrocarbon reserves can be influenced by understanding the evolution and distribution of porosity, but also permeability, related to MHAB.
(iv) Coastal management. MHAB offers eco-friendly solutions to produce biocement in an environmentally friendly way, a process that may aid to reinforce existing marine structures with in-situ occurring MHAB strains and thus may help to combat coastal erosion through biostabilization.

This research highlights the intricate interactions between microbial activity, carbonate mineralogy, morphology, and geochemistry, providing valuable insights for ecological understanding and practical applications in resource management, storage, and conservation. It enhances our knowledge of bacterially mediated alterations in carbonate grains within shallow-marine environments, emphasizing their dual role in mineral precipitation and grain modification. Aerobic heterotrophic bacteria notably promote the formation of Mg-carbonate micrite, influencing porosity evolution during later burial diagenesis. Additionally, bacterial Mg-rich carbonates exhibit distinct and unique chemical biosignatures, particularly carboxylated compounds, which serve as reliable proxies for biological activity and provide critical information about the environmental conditions under which terrestrial and Martian carbonates form. This study opens new avenues for exploring microbial-mediated carbonate formation and underscores its essential role in biogeochemical cycling within natural ecosystems.

## Material and methods

### Bacterial culture experiments

Culture experiments were performed using two moderately halophilic aerobic bacteria strains (MHAB) and two types of carbonate substrates (as described in S1.1). Experiments were incubated for 12 months at 30 °C. The two bacterial strains used for this experiment, *Virgibacillus marismortui* AJ009793 and *Halomonas meridiana* UQM 335, were isolated from the sediment of Brejo do Espinho, a shallow hypersaline coastal lagoon forming Mg-rich carbonates^60^. The bacterial strains are chemoorganotrophic and Gram-negative and solely metabolize organic matter, releasing residual^22–24^ CO_2_, NH_3_, and PO_4_^3−^. These aerobic heterotrophs are known to actively promote Ca and/or Mg carbonate precipitation at ambient surface conditions and are present in modern-day^60,81^ and Arabian Gulf shallow-marine environment^34,42^.

The substrates (10 g each) were sterilized by autoclaving for 15 min at 121 °C before the start of all experiments in this study. The composition of the growth liquid medium is as follows: 0.1% glucose, 0.5% yeast extract, 1% peptone, 3.5% NaCl. The medium was supplied with magnesium and calcium with final Mg:Ca molar ratio of 7. The pH was adjusted to 7.2 by adding 0.1 M KOH. Liquid cultures were carried out in 500-mL Erlenmeyer flasks containing 250 mL of medium. Table 3 shows all the culture experiments (substrates used) with and without cells (control, sterile experiment). Culture experiment Aa3V, substrate composed of ooids, was inoculated with *V. marismortui* cells. Meanwhile, culture experiments Rb3V and Rc3H, substrates composed of skeletal grains, were inoculated with *V. marismortui* and *H. meridiana,* respectively. After incubating for 12 months, the substrates from all the experiments were recovered, washed with distilled water to free them of impurities, and air-dried at 37 °C prior to further analysis.

**Table 3:** Type of carbonate sediment substrate used in each experiment with and without bacteria incubated under aerobic conditions at 30 °C.

Experiment	Substrate	Bacterium	Location
*Aa1*	Ooids	–	Abu Dhabi, UAE
*Rb1*	Skeletal grains	–	Red Sea (SA)
*Rc1*	Skeletal grains	–	Red Sea (SA)
*Aa3V*	Ooids	*V. marismortui*	Abu Dhabi, UAE
*Rb3V*	Skeletal grains	*V. marismortui*	Red Sea (SA)
*Rc3H*	Skeletal grains	*H. meridiana*	Red Sea (SA)

### X-ray diffraction study

Powder X-ray Diffraction (XRD) analyses were made to identify the mineral composition of the substrate samples before and after the experiments using a Bruker X-ray diffractometer model D8 Twin with a LynxEye XE-T detector, using Cu-Kα radiation (λ = 1.5406 Å; operating at 40 kV and 40 mA) at KAUST Core Labs (KSA). Scans were performed of powder samples in the range of 10°–60° 2θ, at a scan rate of 10 s per 0.02°, using a divergence *slit* of 0.6. Qualitative and semi-quantitative phase identification and analysis of the XRD diffractograms were performed using the open-source software package PROFEX^82^ and the Rietveld refinement software package BGMN^83^.

The mole percentage of magnesium was semi-quantitatively calculated from the interplanar d-spacing of the diffraction patterns (*d*_104_) of each carbonate phase using Lumsden equation^84^. Based on published studies^85,86^, we define low-Mg calcite (LMC) as containing less than or equal to 4 mol% of MgCO_3_, high-Mg calcite (HMC) containing between 4 and 30 mol% of MgCO_3_, Ca-rich dolomite (Ca-dol) containing 30–45 mol% MgCO_3_ and stoichiometric dolomite (Dol) from 45–50 mol% MgCO_3_.

### Elemental composition

All samples were powdered, and an aliquot of 100 mg was dissolved with 2 M HNO_3_ and sequentially diluted twice with 5% HNO_3_ to a final dilution factor of 100.000. Experimental solutions were diluted twice as well to a total dilution factor of 100. Samples were measured with an Inductively Coupled Plasma Optical Emission Spectrometry (ICP-OES-Varian 720-ES) at the Earth Sciences Department, Vrije Universiteit Amsterdam, The Netherlands. Elemental concentrations of Ca^2+^ and Mg^2+^ were reported as weight percentage (wt %) and Sr^2+^ in parts per million (ppm) for the substrates. For the analyzed experimental solutions, Ca^2+^ and Mg^2+^ are reported in mmol, while Sr^2+^ is in ppm.

### Stable isotope analysis

Stable carbon and oxygen isotopic compositional analysis of powdered carbonate samples was performed using a Finnigan™ MAT253 mass spectrometer and Gasbench II preparation system at the Earth Sciences Department, Vrije Universiteit Amsterdam, The Netherlands. The isotope data were reported in the standard delta (δ) notation per thousand (‰) relative to the international Vienna Pee Dee Belemnite (VPDB). The sample size was corrected using the internal standard (VICS), while the international IAEA-603 was measured as a control standard. The long-term standard deviation of the routinely analyzed in-house standard is < 0.1‰ (1σ) for both carbon and oxygen isotope ratios.

The stable oxygen and carbon isotopic composition of the dissolved inorganic carbon (DIC) were measured on a Thermo Finnigan Gasbench II interfaced with a Thermo Finnigan Delta + mass spectrometer (Thermo Finnigan MAT253) at the Stable isotope laboratory at the Vrije Universiteit Amsterdam. Oxygen isotopes were measured in duplicate alongside four in-house water standards calibrated to international standards. Three water standards were used for sample calibration, and KONA was used to determine measurement accuracy. For the calibration of ^13^C values, two carbonate standards were used. A sodium bicarbonate solution was prepared to monitor DIC measurement precision. KONA’s ^18^O SD is < 0.1‰, and ^13^C DIC’s SD is < 0.15‰.

### Solid-state NMR analysis

Solid-state nuclear magnetic resonance (NMR) analyses were performed on the substrates recovered from MHAB cultures and control experiments to understand their compositional and structural characteristics at the molecular level. One-dimensional ^13^C cross-polarization (CP) and direct polarization (DP) magic angle spin (MAS) solid-state nuclear magnetic resonance (NMR) spectra were recorded on Bruker ADVANCE III™ spectrometers operating at 600 MHz resonance frequencies for 1 h. 600 MHz experiments employed a conventional double resonance 3.2 mm CP MAS probe. In all cases, dry nitrogen gas was utilized for sample spinning to prevent degradation of the samples. NMR chemical shifts are reported concerning the external references TMS and adamantane. The following sequence was used: 900 pulses on the proton (pulse length 2.4 s), then a cross-polarization step with a contact time of 2 ms, and finally, acquisition of the ^13^C signal under high-power proton decoupling. The delay between the scans was set to 4 s to allow the complete relaxation of the ^1^H nuclei, and the number of scans ranged between 5000 and 10,000 for ^13^C and 8 for ^1^H. An exponential apodization function corresponding to a line broadening of 80 Hz was applied prior to the Fourier transformation.

### Scanning electron microscopy study

Optical and electron microscopy of the carbonate sediments (substrates) were performed for petrographic, morphological, and compositional analysis before and after the bacterial culture experiments. Petrographic thin sections of original sediments were imaged using a Leica DM2700 P microscope at 2.5×, 4×, and 10× magnifications using plane-polarized light. We performed a high-resolution SEM analysis of six samples recovered from bacterial cultures and control experiments (Table 1). Given the small quantities of sediment samples available after the culture experiments, a unique sample preparation technique was used with mm-scale sample vials for resin casting. We used a four-component low-viscosity resin kit was deployed to embed dry powder samples. The resin casts were placed in a vacuum oven at 65 °C for 24 h to cure. The cured samples were then polished mechanically, followed by ion polishing, to obtain the ideal sample surface suitable for SEM analysis using Secondary-Electron (SE), Back-Scatter Electron (BSE), and Energy Dispersive Spectral (EDS) detectors. The polished samples were then sputter-coated with a 4 nm thick Platinum layer prior to SEM imaging. Secondary electron micrographs were acquired with platinum-coated samples (see SI) using a Thermofisher FESEM TeneoTM, at KAUST, operated at 20 kV and current varying from 1.6 to 3.2 nA to minimize artifacts caused by the charging effect. SE, BSE, and EDS point and mapping analysis were performed at magnifications ranging from 50× to 10,000×.

## Supplementary Information

Supplementary Information.

## Data Availability

The data underlying this article are available in *the Mendeley Data repository* (https://data.mendeley.com), at 10.17632/pt6ysc8v6d.1.
