# Supplementary material for: The hidden role of heterotrophic bacteria in early carbonate diagenesis
Source: Sci Rep. 2025 Jan 2;15:561. doi: 10.1038/s41598-024-84407-y (PMC11696100; doi:10.1038/s41598-024-84407-y)
Supplement: Supplementary file 1 — Supplementary Information. [file 41598_2024_84407_MOESM1_ESM.pdf]

Supplementary Materials for

The hidden role of heterotrophic bacteria in early carbonate diagenesis

Mónica Sánchez-Román[1*], Viswasanthi Chandra[2,3], Sebastian Mulder[1,4], Camila Areias[1], John Reijmer[1] and Volker Vahrenkamp[2]

*Corresponding author. Email: m.sanchezroman@vu.nl or sanchezromanmonica@gmail.com

Material and Methods

Field sediment substrate collection

We used Holocene sediment samples collected from two shallow marine locations in the Middle East: 1) the ebb delta ooid shoals of north-east of Abu Dhabi in the Arabian Gulf (Aa) (24°36'15.61"N; 54°28'11.64"E) and 2) shallow intertidal carbonate sands from the north-west of Saudi Arabia in the Red Sea (Rb) (22°19'23.10"N; 38°51'22.96"E). The climate along the coast of Abu Dhabi varies between arid and humid. The average humidity values range from 40% during the day to 90% at night. A more comprehensive discussion of the depositional environment and sediment distributions of the Holocene carbonates of Abu Dhabi can be found in [1]. Water temperatures vary from 14° to 40° C. The sediments in our sampling location are predominantly shallow-water carbonate sediments, including ooids, grapestones, pellets, muds, and bioclastic debris. The samples collected for this study were obtained from the sub-marine surface of the exposed ooid shoals' area, characterized by a tidal delta depositional setting and a high-energy environment. The Holocene sediments collected from the Red Sea originate from a beach on Shaybarah island, located southeast of the Al Wajh platform in the northeastern Red Sea of Saudi Arabia. The climate here is arid, with low precipitation rates between 1 cm year^{-1} and 20 cm year^{-1} and high evaporation rates of up to 2 m year^{-1}. The average humidity and

temperature at the Red Sea location are over 30% and 30° C in summer and 50% and 23° C in winter, respectively [2,3].The depositional setting can be locally characterized as shallow intertidal, and the sediments are mainly composed of benthic foraminifera, reef debris, gastropods, bivalves, peloids, and echinoderm fragments.

Petrography of original sediments

Thin-section petrography analyses confirmed that the Arabian Gulf sediments (Aa) used in this study primarily comprise ooids and peloids, characterized as spherical and ovoid grains (Fig. S1A). The sediments are moderately sorted, with an average grain size between 50 and 400 μm. The ooid grains are identified by their concentric aragonite laminations around the solid nuclei, which are primarily skeletal fragments and, less commonly, detrital quartz. The peloids, on the other hand, were differentiated based on their lack of laminations. The outer rims of the ooid and peloid grains are heavily micritized and characterized by extensive microborings, partially filled by aragonite, as indicated by XRD (Table 2). Micritization was also observed within the grains, varying in extent from the outer laminations toward the nucleus. The sediments from the Red Sea (Rb, Fig. S1B) are mixed bioclastic carbonate grains, mainly composed of coral fragments, benthic foraminifera, bivalves, gastropods, and other skeletal fragments. The sediment grains are well sorted with an average grain size between 200 to 800 μm (Fig. S1B). Micrite associated with partially to filled microborings was also observed in these sediments, especially in the benthic foraminiferal skeletons.

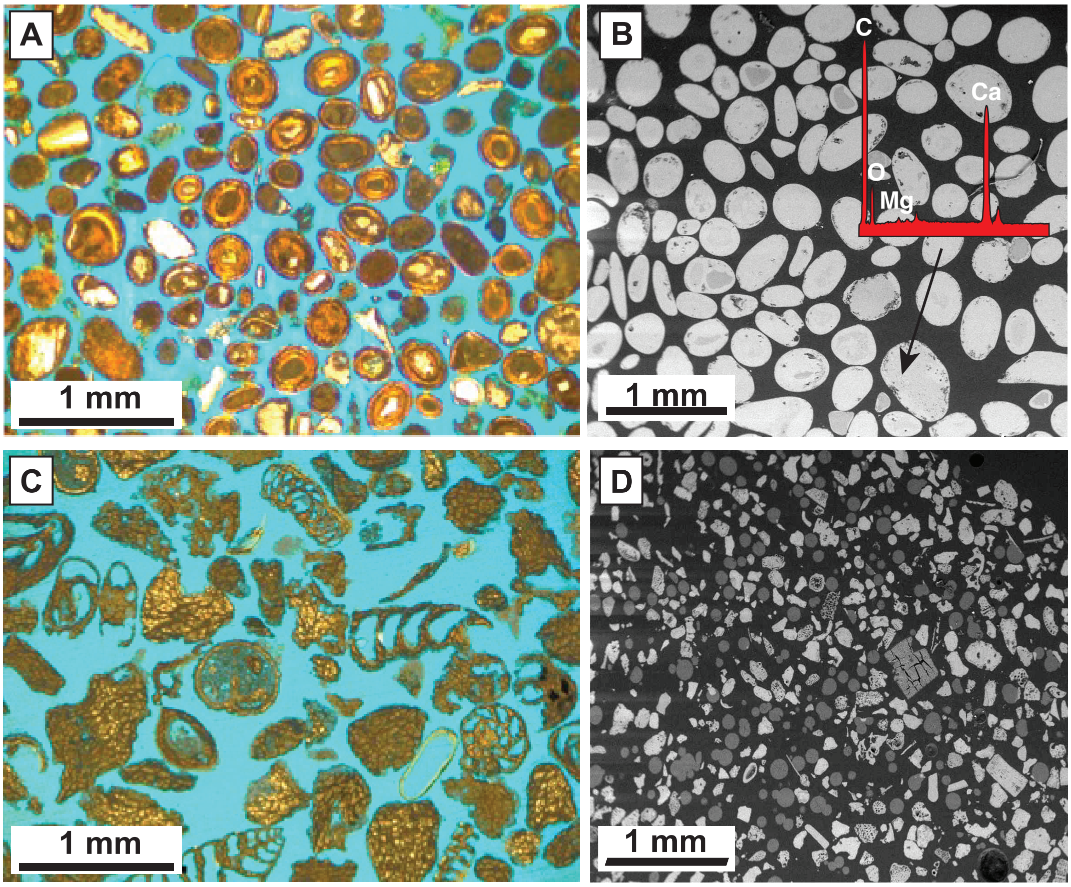

Figure S1. *Thin section micrographs imaged using plane-polarized light illustrate the components of sediment samples used in this study. (A) Arabian Gulf intertidal sediments (Aa) primarily comprise ooids and peloids. (B) SEM overview and EDS indicate that the grains generally comprise aragonite with low or no Mg content. (C) The Red Sea inner lagoon sediments (Rb) are a mixture of bioclastic grains, mainly gastropods, bivalves, benthic foraminifera, and reef debris. (D)*

Geochemical study – Mineral saturation indices

The activity of dissolved species and the degree of saturation in the initial solutions assayed were determined using the geochemical computer program PHREEQC, version 2 [4].The results from PHREEQC are presented in terms of the saturation index (SI) for each predicted mineral. SI is defined by SI=log (IAP/ Ksp), where IAP is the ion activity product of the dissolved constituents and Ksp is the solubility product for the mineral. Thus, SI>0 implies

supersaturation with respect to the mineral, whereas SI<0 means undersaturation. All calculations were performed applying the following starting values in the media (g/l): Mg^{2+}=2, Ca^{2+}= 0.44, Na^{2+}=14, Cl^{-}=21.23, P=0.15 ($PO_4{}^{3-}$=0.46), $NH_4{}^{+}$=1.73. The values of Na^{+}, Cl^{-}, P and $NH_4{}^{+}$ correspond to the addition of NaCl=35 g/l, proteose peptone=5 g/l and yeast extract=10 g/l. Total nitrogen in the culture media was determined by Kjeldhal's method, while total phosphorus was determined colorimetrically in the nitrogen digests, generating the phosphomolibdate complex [5]. Also, we should note that the initial CO_2 value used in these geochemical calculations is 0.004 mg/l assuming that atmospheric CO_2 was in equilibrium with saline medium.

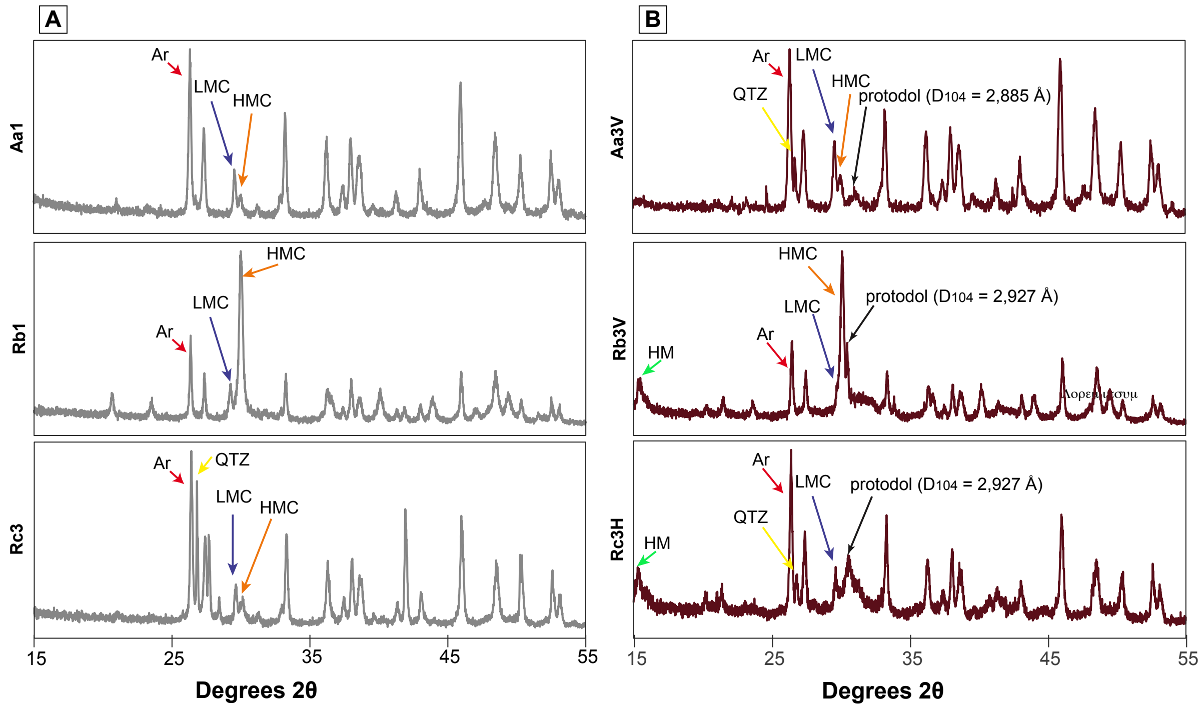

Figure S2. Representative X-ray diffraction patterns (XRD) of bulk sediment samples. (A) Sediments from *V. marismortui* (Aa3V and Rb3V) and *H. meridiana* culture and sterile substrates from control (Aa1, Rb1 and Rc1) experiments recovered after one year of incubation at 30° C (B). Reference peaks of major components correspond to aragonite (Ar), High Magnesian Calcite (HMC), Protodolomite (protodol), and Hydromagnesite (HM). Other reference peaks correspond to minor components, including quartz (QTZ) and Low Magnesium Calcite (LMC). Note: The main peak (104) of protodolomite (disordered dolomite) precipitates is at at 30.968° (2-theta) and d-spacing (104) = 2.885Å for Aa3V, and at 30.520° (d_{104} = 2.927Å) for Rb3V and Rc3H samples; while 104 peak of ordered dolomite is at 30.86° (d_{104} = 2.897Å) (data taken from American Mineralogist Crystal Structure Database). The d_{104} of protodolomite precipitates from MHAB falls within the range of previously reported disordered dolomite from solutions containing ethanol and calcite seeds (d_{104} = 2.916, 2.918, 2.936, 2.940) [6] and from bacterial experiments (d_{104} = 2.907, 2.904, 2.916) [7].

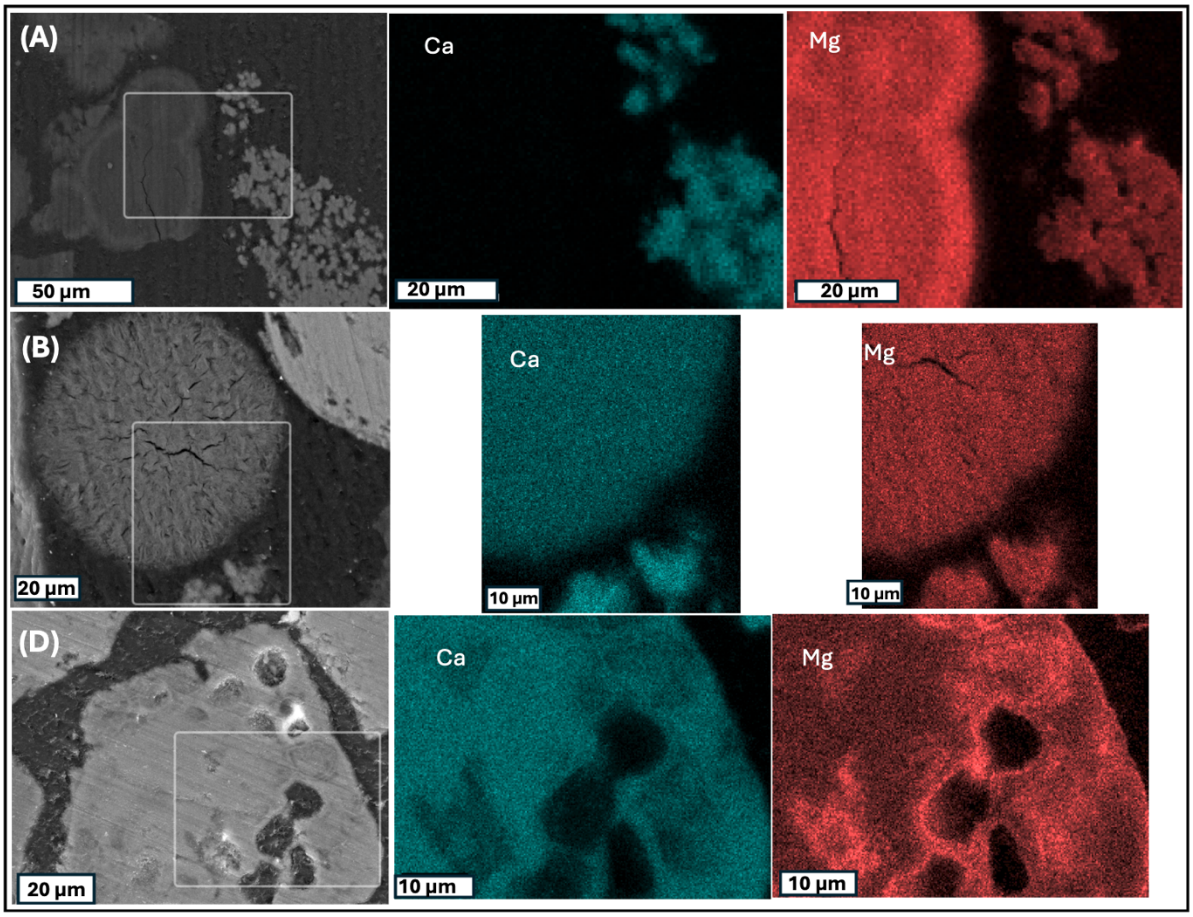

Figure S3. SEM backscatter images of Mg-rich carbonate precipitates from MHAB culture experiments. (A) hydromagnesite ovoidal spherulites and aggregate of protodolomite spherulites (Aa3V), (B) protodolomitic sphere (Rb3V), and (C) a grain enriched in Mg, particularly along the borders of the pores and the outer rim (Rc3H).

Table S1. Saturation index values (SI) for different minerals in the medium assayed.

Mineral phase	SI
Aragonite, $CaCO_3$	-6,5
Calcite, $CaCO_3$	-6,2
Dolomite, $CaMg(CO_3)_2$	-11,2
Halite, NaCl	-2,4
Hydromagnesite, $Mg_5(CO_3)4(OH)_2•4H_2O$	-35,4
Hydroxyapatite, $Ca_5(PO_4)_3OH$	7,6

Results of the geochemical computer program PHREEQC. SI is defined by SI=log (IAP/Ksp), where IAP is the ion activity product of the dissolved mineral constituents in a solubility product (Ksp) for the mineral. Thus, SI >0 implies supersaturation with respect to the mineral, whereas SI <0 means undersaturation. Note: All calculations were performed applying the following starting values in the media (g/l): Mg^{2+}=2, Ca^{2+}=0.44, Na^{2+}=14, Cl^{-}=21.23, P=0.15 (PO_4^{3-}=0.46), NH_4^{+}=1.73.

Table S2. Full set of measurements for carbon and oxygen isotopic composition of substrates recovered from all the experiments. Results are reported in parts per thousand (‰) relative to VPDB.

	**$\delta^{13}C$ (‰ vs VPDB)**	**Cstd**	**$\delta^{18}O$ (‰ vs VPDB)**	**Ostd**
Aa1	3.97	0.24	0.24	0.06
	3.98	0.27	0.27	0.05
	3.94	0.24	0.24	0.06
	4.33	0.05	0.28	0.06
	3.78	0.03	-0.01	0.03
Aa3V	-9.14	0.03	-4.87	0.05
	-8.87	0.05	-4.89	0.07
	-8.57	0.04	-4.95	0.04
	-11.55	0.03	-5.59	0.08
Rb1	2.62	0.05	-1.11	0.06
	1.92	0.05	-1.82	0.1
	2.08	0.05	-1.6	0.05
Rb3V	-2.82	0.07	-2.74	0.07
	-2.71	0.06	-2.75	0.05
	-10.18	0.06	-5.01	0.08
	-8.81	0.04	-4.38	0.02
Rc1	1.11	0.05	-0.35	0.05
	1.13	0.05	-0.33	0.04
	-1.47	0.05	-1.84	0.03
	-1.2	0.04	-1.03	0.04
Rc3H	-3.61	0.07	-2.51	0.06
	-5.02	0.06	-2.55	0.05
	-4.36	0.06	-2.6	0.05
	-6.15	0.06	-2.82	0.09
	-4.26	0.06	-2.32	0.06

References

[1] Kendall, C. G. St. C. & Alsharhan, A. S. Coastal Holocene carbonates of Abu Dhabi, UAE: depositional setting, sediment distribution, and role of cyanobacteria in micritization. Quaternary carbonate and evaporite sedimentary facies and their ancient analogues: A Tribute to Douglas James Shearman 205–219 (2010).

[2] Bruckner, A., Rowlands, G., Riegl, B., Purkis, S., Williams, A. & Renaud, P. Atlas of Saudi Arabian Red Sea Marine Habitats, 2nd ed. Panoramic Press (2012).

[3] Petrovic, A., Ariza Fuentes, M., Putri, I., Yahaya, L.N., Khanna, P., Purkis, S.J. & Vahrenkamp, V. Holocene sediment distribution in the Al Wajh platform lagoon (northern Red Sea, Saudi Arabia), a modern analogue for large rift basin carbonate platforms. Sedimentology 69, 1365–1398 (2022).

[4] Parkhust DL & Appelo CAJ (1999) User's guide to PHREEQC (Version 2) – a computer program for speciation, batchreaction, one-dimensional transport, and inverse geochemical calculations. Water-Resources Investigations Report 99–4259, US Geological Survey, Denver, CO.

[5] Page AL, Miller RH & Keeny DR, (eds) (1982) Methods of Soil Analysis, Part 2, Chemical and Microbiological Properties. American Society of Agronomy and Soil Science Society of America, Madison, WI.

[6] Fang Y, Zhang F, Farfan GA, and Xu H. Low-Temperature Synthesis of Disordered Dolomite and High-Magnesium Calcite in Ethanol–Water Solutions: The Solvation Effect and Implications. ACS Omega 27 (1), 281-292 (2022) DOI: 10.1021/acsomega.1c04624.

[7] Liu, D., Yu, N., Papineau, D., Fan, Q., Wang, H., Qiu, X., She, Z., Luo,G. The catalytic role of planktonic aerobic heterotrophic bacteria in protodolomite formation: Results from Lake Jibuhulangtu Nuur, Inner Mongolia, China, *Geochim. Cosmochim. Ac.* **263**, 31-49 (2019).
